# Establishment and Polymorphism Analysis of SNP Markers in the Gynogenic Blunt Snout Bream

**DOI:** 10.3390/biology15020188

**Published:** 2026-01-20

**Authors:** Ping Wu, Yuhuan Wei, Siyao Weng, Mingguang Hu, Jiaxing Li, Wenxuan Tang, Lei Zhang, Qinbo Qin, Ting Yi, Wuhui Li, Min Tao, Chun Zhang, Qizhi Liu, Shaojun Liu

**Affiliations:** 1 Engineering Research Center of Polyploid Fish Reproduction and Breeding of the State Education Ministry, College of Life Sciences, Hunan Normal University, Changsha 410081, China; wuping@hunnu.edu.cn (P.W.);; 2 Yuelushan Laboratory, Changsha 410128, China; 3 Hunan Province Fish Seed Breeding Technology Co., Ltd., Shaoyang 422499, China; 4 Hunan Yuelu Mountain Science and Technology Co., Ltd. for Aquatic Breeding, Changsha 410081, China

**Keywords:** GBSB, BSB, SNP marker, molecular marker assisted breeding

## Abstract

The blunt snout bream (*Megalobrama amblycephala*, BSB) is a freshwater economic fish with Chinese characteristics, and its genetic characteristics have unique value for studying fish evolution. The gynogenetic blunt snout bream (GBSB) obtained through distant hybridization between cross-order species, which showed a faster growth rate than the female parent, but its appearance is similar to that of BSB and is difficult to distinguish. Therefore, by comparing the transcriptome sequencing data of BSB and GBSB (SRA number: PRJNA893089, not released yet), we identified 30 SNPs associated with genes related to muscle growth, protein synthesis, and glycolysis that are unique to GBSB. Through multi-sample PCR detection and sequencing analysis, 16 SNPs with stable differences in GBSB and BSB were obtained. The polymorphism analysis of 16 SNP sites showed that 9 SNP sites were polymorphic in GBSB, which could be used to identify GBSB and its female parent, BSB. In addition, the 9 SNP sites are located in the *myoz1a* (*myozenin 1a*) gene, which is related to muscle development, and may provide insights for further study of muscle growth regulation. Therefore, this study provides candidate marker resources for GBSB germplasm resource identification and molecular marker-assisted breeding, which is beneficial for improving the efficiency and reliability of selection and breeding work.

## Simple Summary

Blunt snout bream is an economically important freshwater fish in China, and the gynogenetic blunt snout bream (GBSB) exhibits faster growth and improved nutritional quality, yet is visually indistinguishable from the common blunt snout bream (BSB), posing challenges for breeding and aquaculture management. This study aimed to develop molecular markers for the accurate identification of GBSB. Through comparative transcriptome analysis of muscle tissue, 30 candidate SNP markers were identified, with nine consistently polymorphic markers specific to GBSB being validated in 30 individuals per group. These markers are located in the muscle development-related *myoz1a* gene, which may provide insights into the growth advantages of GBSB. The findings offer a practical molecular tool for aquaculture breeding, supporting marker-assisted selection and the sustainable development of fish farming.

## 1. Introduction

Megalobrama amblycephala, commonly known as the blunt snout bream (BSB, 2n = 48), belongs to the Cyprinidae and is an economically important freshwater fish species in China. Due to overfishing, wild populations of BSB have been severely depleted, and artificial cultivation has become the predominant mode of production [1]. Genetic improvement of existing BSB populations to develop superior strains with desirable traits is crucial because this will contribute to the sustainable development of aquaculture in China [2,3].

Gynogenesis is a powerful breeding technique that can speed up selective breeding in various populations, while also altering the genetic homozygosity of the species, so that the hybrid progeny may show heterosis in terms of growth rate and disease resistance [4,5]. It has been extensively applied across numerous fish species, including the Japanese crucian carp [6], turbot [7], and red crucian carp [8]. Previous studies have shown that a small amount of paternal genetic material will be integrated into the gynogenesis to produce progeny during the process of heterologous sperm induction, and this phenomenon is called “micro-hybrid” [9,10]. These paternal fragments may contribute to genetic variation and provide a basis for SNP marker development, which can not only be used as effective molecular markers to distinguish female diploid from ordinary diploid [11,12], but it is also highly likely that the progeny will acquire certain favorable traits. Thus, the phenomenon is called the “heterospermic effect”. In previous studies, we have cultivated the gynogenesis blunt snout bream (GBSB) with a higher growth rate and better muscle nutrition characteristics than the female parent by gynogenetic technology [3,13]. However, it is difficult to distinguish GBSB from its female parent in appearance. At present, there is no research on the markers used to distinguish the two fish species, so it is necessary to develop specific markers for GBSB. However, current marker-based identification still faces the limitations of a limited number of markers, insufficient population specificity, and insufficient cross-group verification in fish breeding.

Single-nucleotide polymorphisms (SNPs) belonging to the third generation of genetic marker technology refer to the polymorphism of DNA sequences caused by the variation in a single base at the genome level. As the most important type of DNA variation in the genome, they play a crucial role in animal genetic research [14,15,16]. SNP markers are widely used in molecular-assisted breeding and germplasm identification due to their widespreadness, representativeness, genetic stability, and easy automation of analysis [17,18,19,20,21,22,23,24]. In fish, the development and application of SNPs have been reported in several studies, such as blunt snout bream [1], aucha perch [25], catfish [26], turbot [27], etc. However, SNP markers have not yet been reported in GBSB. Therefore, based on the high-throughput sequencing data of GBSB transcriptome and previous studies, this study aimed to develop GBSB SNP markers to provide candidate marker resources for GBSB germplasm resource identification and molecular marker-assisted breeding.

## 2. Materials and Methods

### 2.1. Ethics Statement

All experimental procedures involving animals were conducted in accordance with the institutional animal care and use guidelines approved by Hunan Normal University.

### 2.2. Gynogenesis and Fish Rearing

The experiment was conducted at the Xuefeng Mountain Fish Breeding Base located in Wugang, Hunan Province, China. Parental stocks of blunt snout bream (BSB) and Chinese perch (Siniperca chuatsi, CP) were sourced from the Hunan Provincial Institute of Fisheries Science. Between May and July 2021, twenty sexually mature individuals of each species were randomly selected as broodstock. Females received a single intramuscular injection containing domperidone (1.0–2.0 μg/kg), LHRH-A2 (10.0–12.5 μg/kg), and HCG (200–250 IU/kg). Males were administered a single injection of domperidone (2.0–5.0 μg/kg) and LHRH-A2 (2.0–5.0 μg/kg). After 8–14 h, sperm were collected by gently pressing the abdomen of the CP, and mature eggs were obtained by squeezing the abdomen of the BSB. The eggs and sperm were combined for fertilization, and the resulting fertilized eggs were transferred to a clean incubator with water temperatures maintained at 22 °C–25 °C. The fertilization rate of gynogenetic blunt snout bream was 22.28%, and the hatching rate was 2.92%. The hatched GBSB fry were reared in ponds at a stocking density of 400 individuals per acre and fed a high-protein diet for five months. The specific production process and feeding situation of GBSB can be referred to [28,29]. It was confirmed that the DNA content, chromosome number (n = 48), and 5S rDNA sequence (188 bp and 376 bp) of GBSB were consistent with those of the female parent, which was previously consistent with Wang et al. (2022) [30]. Weight references for the three fish species follow Wu et al. (2024) [13]. For subsequent analysis, thirty age-matched fish from both BSB and GBSB groups were randomly sampled. White muscle tissue specimens (1 cm × 0.5 cm × 0.5 cm) were dissected from below the dorsal fin of each individual for experimental use.

### 2.3. Screening of the Transcriptome SNP

Total RNA was extracted from the muscle tissues of BSB and GBSB, and the cDNA library was established and sequenced with Illumina Novaseq (Novogene Co., Ltd., Beijing, China). Fastp 0.19.7 software was used to evaluate the quality of the original sequencing data, check the sequencing error rate, GC content, connector contamination, etc. Filter low-quality data: Remove low-quality bases (Q < 20), adaptor sequences, and short reads (e.g., <50 bp) by Trimmomatic 0.36. The Megalobrama amblycephala reference genome (Genome assembly ASM1881202v1; NCBI Assembly ID: GCF_018812025.1) was used for SNP calling. The obtained SNP was annotated by the SnpEff 4.3 software, and the obtained SNP was subjected to quality control and filtering. Individual SNPs were detected using GATK 4.1.1.0 software [31] (Parameters: gcpHMM 10-stand_emit_conf 10-stand_call_conf 30). Filtering false positives was based on the following criteria to filter low confidence sites: sequencing depth (Depth < 10×); quality value (QUAL < 30); chain preference (such as FS > 60); allele frequency (MAF < 0.05); heterozygosity abnormalities (such as not meeting the Hardy–Weinberg equilibrium). In order to reduce the error rate of SNP detection, the following criteria were used for filtering: SNP: QD < 2.0, FS > 60.0, MQ < 30.0, HaplotypeScore > 13.0, MappingQualityRankSum < −12.5, ReadPosRankSum < −8.0. The filtered SNP was used for further data analysis.

### 2.4. Preparation of the DNA Samples

Thirty-six individuals each from the BSB and GBSB groups were randomly selected. Genomic DNA was extracted using a tissue DNA extraction kit (TaKaRa, Dalian, China). The concentration and purity of the extracted DNA were measured using a NanoDrop ultraviolet spectrophotometer, and its integrity was assessed by agarose gel electrophoresis. Samples meeting the quality criteria were stored at −20 °C for subsequent experimental procedures.

### 2.5. Verification of the SNP Marker Sites

In order to verify the credibility of SNP markers, 30 SNP loci related to our research objectives were selected according to the function of the corresponding genes, primers were designed by Primer5.0 and synthesized by Sangon Biotech (Shanghai, China). The DNA samples of 6 individuals per fish species were mixed in equal amounts as the template for detection. The DNA mixing strategy used in our study was limited to the preliminary screening stage of SNP markers, and the purpose was to efficiently identify the differential sites between BSB and GBSB populations. All subsequent polymorphism parameter calculations (including PIC, H, Ne, MAF, and HWE tests) were based on independent genotyping data from 30 individuals [32]. Alleles in mixed samples are only used as preliminary indicators of presence, while quantitative analysis, such as frequency estimation and heterozygosity calculation depend entirely on genotype data at the individual level, which ensures the scientific rigor of polymorphism analysis. To efficiently screen a large number of SNP loci, we used a pooling strategy for the preliminary validation step. Referring to the method of Zhou et al., six individual DNA samples of each fish were evenly mixed as the detection template [25]. The PCR reaction was 20 μL: 3 μL of DNA template, 10 μL of 2× ES Taq Master Mix, 0.8 μL of upstream primers, 0.8 μL of downstream primers, 0.8 μL, and 5.4 μL of ddH_2_O (Vazyme, Nanjing, China). The PCR reaction procedure was as follows: pre-denaturation at 94 °C for 5 min; Denaturation at 94 °C for 30 s, annealing temperature (depending on the specific primer Tm value) for 30 s, extension at 72 °C for 30 s, a total of 35 cycles. Finally, extend at 72 °C for 7 min and store at 4 °C. PCR amplification products were detected by 1.5% agarose gel electrophoresis and genotyped using next-generation sequencing technology, which was supported by Sangon Bioengineering (Shanghai, China) Co., Ltd. Subsequently, according to the sequencing results, the remaining 30 fish were tested to expand the sample.

### 2.6. SNP Marker Function Analysis

The SNP sites in the sequencing results were compared using DNAMAN 8.0 software (LynnonBiosoft, San Ramon, CA, USA). Functional annotations were performed on the selected SNP sites through NCBI’s BlastP (https://blast.ncbi.nlm.nih.gov/Blast.cgi, accessed on 1 May 2025) and UniProt (https://www.uniprot.org/uniprotkb, accessed on 1 May 2025) databases. Open reading frames (ORFs) were identified using ORF Finder and cross-verified against NCBI databases to ensure accurate selection of ORFs, thereby determining the positions of SNP sites within genetic codons.

### 2.7. Genetic Polymorphism Analysis

The typing results of SNP sites were processed by POPGEN32 (version 1.32) software, and the effective number of alleles (Ne) and heterozygosity (H) were counted for each site [33]. The genotype distribution and frequency of each point and the minor allele frequency (MAF) were also counted. PIC_CALC (0.6) was used to calculate the polymorphism information content (PIC) of each point.

### 2.8. Statistical Analysis

The coupling probability (matching probability, MP) was calculated using the formula [34].

$$MP=\prod_{k=1}^{m}(\sum_{i=1}^{n}\left(P_{k_{i}}^{2}\right)+\sum_{i=1}^{n}\sum_{j=i+1}^{n}{\left(2P_{k_{i}}P_{k_{j}}\right)}^{2})$$

In this formula, m is the number of sites; n is the number of alleles at site k; and $P_{k_{i(j)}}$ is the frequency of alleles i and j at site k.

## 3. Results

### 3.1. Analysis of Polymorphic Sites and Screening of Core SNP Sites in Transcriptome

**Figure 1:**
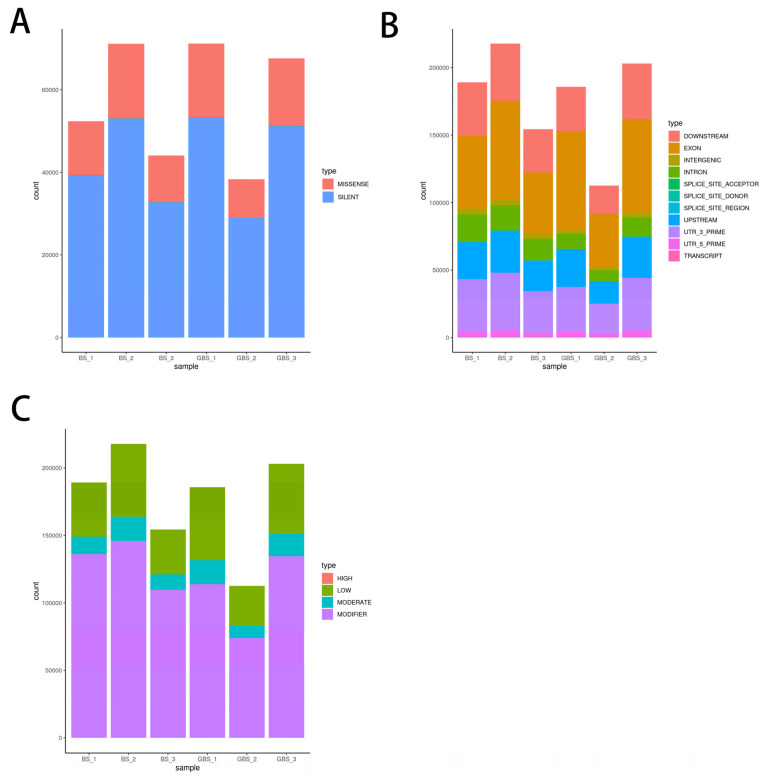
SNP distribution and statistical analysis. (**A**) Distribution of SNP mutation types. The proportion of silent mutations was the highest (74.36–75.81%), indicating that most SNPs did not change the protein sequence. Missense mutations (24.17–25.57%) may affect protein function. (**B**) Distribution of SNP region types. SNP is mainly distributed in the coding region, indicating that the variation may directly affect the gene function. SNP in the 3′ UTR region may affect mRNA stability or translation efficiency. (**C**) Distribution of SNP impact types. The modification’s effect accounted for the highest proportion (61.25–71.94%), suggesting that these SNPs may affect the phenotype by regulating gene expression.

**Figure 2:**
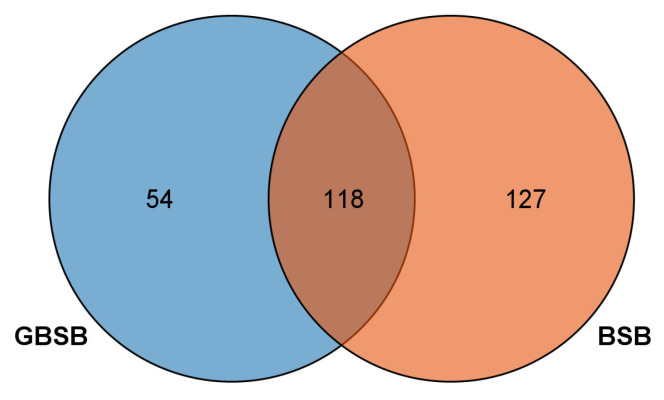
The Venn plot of GBSB-specific SNP sites corresponds to gene screening. The blue circle shows the number of specific genes in GBSB, the orange circle shows the number of BSB, and the brown part in the middle shows the genes shared by both. Details are in Table S4. Gene screening focuses on genes related to growth and metabolism, providing a basis for subsequent SNP functional analysis.

Filter out low-quality sequencing data from the sample genome resequencing data. Finally, the obtained high-quality data were compared with the reference genome of blunt snout bream (GCF_018812025.1). The average alignment rate was 96.25%, and the average sequencing depth was 6.33× (Table 1). After quality control and filtration, 332,974 high-quality loci were finally obtained for subsequent analysis, of which 273,234 were SNP loci, and 59,740 were INDEL loci. By screening transcriptome data, it was found that there are multiple SNP sites that differ between the GBSB and BSB (The raw transcriptome data are stored in the Sequence Read Archive (SRA) of the National Center for Biotechnology Information (NCBI), with access number PRJNA893089 (not released yet) [13]. The SNP mutation spectrum showed that the main type of SNP mutation was silent mutation (74.36–75.81%), followed by missense mutation (24.17–25.57%), and a small number of nonsense mutations (0.01–0.09%) (Figure 1A, Table S1). In addition, the results showed that SNP sites were mainly distributed in the coding region of exons, followed by 3′ UTR and downstream regions (Figure 1B, Table S2). Among the SNP sites distributed in the gene coding region, some of them lead to missense mutation, deletion, insertion, and frame shift mutation of amino acids in the gene coding proteins, which may affect the biological function of some genes in BSB and GBSB. In terms of the impact of SNP mutation, modification accounted for the largest proportion, with a range of 61.25–71.94% (Figure 1C, Table S3). According to the results of transcriptome sequencing, SNPs with coverage greater than 500 were searched, and 245 genes corresponding to SNPs were screened in BSB, 172 genes in GBSB, and 54 genes specific to GBSB were screened. Among these genes, 10 genes related to growth and metabolism were screened according to their functions (including 47 SNP sites, among which 17 were upstream gene mutations, and the corresponding upstream gene SNP sites all existed in the remaining 30 SNP sites) (Figure 2, Table S4).

### 3.2. Validation and Analysis of Core SNP Sites

**Figure 3:**
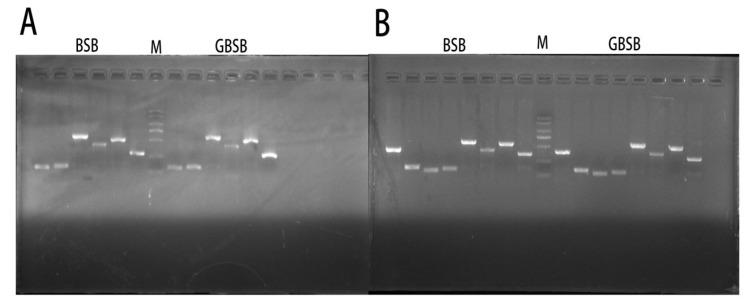
The amplification results of 11 primer pairs were obtained by mixing equal amounts of DNA from 6 individuals of each fish species (BSB and GBSB) as templates. M indicates standard DNA marker, and the bands from top to bottom are 2 kb, 1 kb, 750 bp, 500 bp, 250 bp, and 100 bp, respectively. (**A**) From left to right are BSB *myoz1a-1*, *myoz1a-2*, *myoz1a-3*, *myoz1a-4*, *actn3a*, *klhl41b*, DNA marker, GBSB *myoz1a-1*, *myoz1a-2*, *myoz1a-3*, *myoz1a-4*, *actn3a*, *klhl41b*. (**B**) From left to right are BSB *pgam2-1*, *pgam2-2*, *eef2l2*, *myoz1a-2*, *myoz1a-3*, *myoz1a-4*, *eno3*, *atf4a*, DNA marker, GBSB *pgam2-1*, *pgam2-2*, *eef2l2*, *myoz1a-2*, *myoz1a-3*, *myoz1a-4*, *eno3*, *atf4a*.

**Figure 4:**
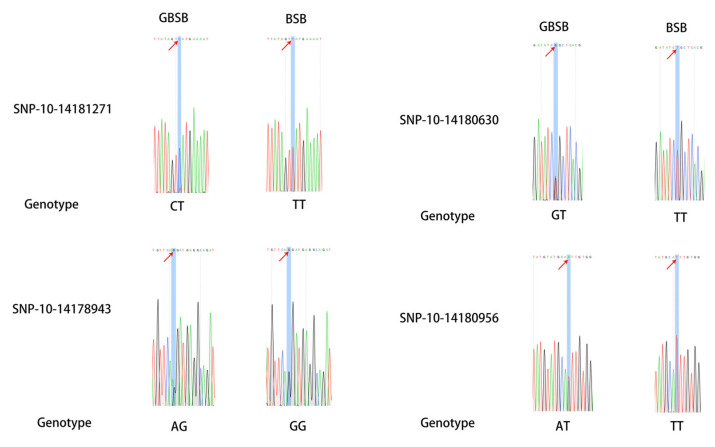
Sanger-seq verification results of some core SNP sites. The base difference between BSB and GBSB at the same position provides direct evidence for population-specific molecular markers. The location of key sites with red arrows in the figure.

**Figure 5:**
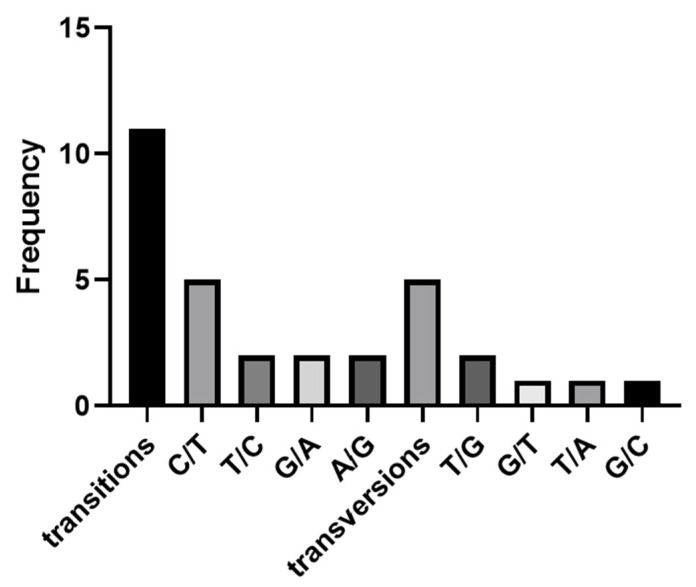
Distribution of SNP variants. The transition was dominant (11/16), which was consistent with the general rule of SNP variation in vertebrates. Transversion was less (5/16), which may be related to the preference of mutation mechanism.

**Figure 6:**
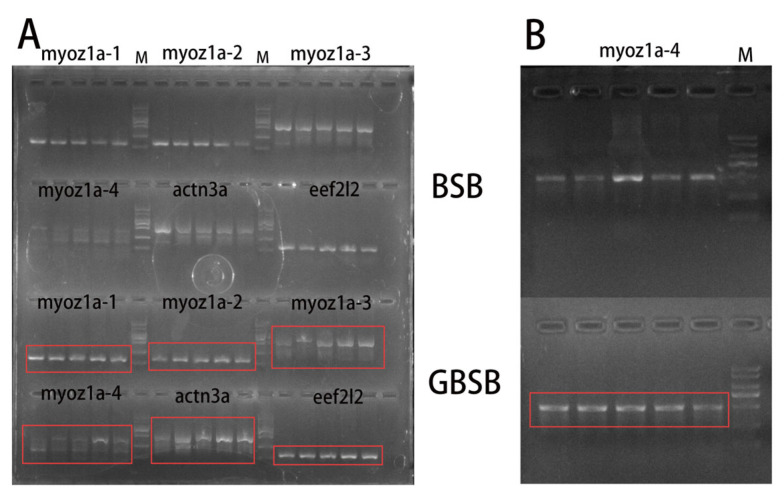
Partial amplification results of 6 primer pairs corresponding to GBSB-specific SNP loci. (**A**) Amplification map of *myoz1a-1*, *myoz1a-2* and *myoz1a-3* gene SNP loci; (**B**) Amplification map of *myoz1a-4* gene loci. M indicates standard DNA marker, and the bands from top to bottom are 2 kb, 1 kb, 750 bp, 500 bp, 250 bp, and 100 bp, respectively. The red box marked the band corresponding to the GBSB-specific SNP locus. The size of the band was consistent with the expectation to ensure the accuracy of subsequent genotyping.

In order to verify the reliability of SNP markers, 6 individuals from each of BSB and GBSB were mixed in equal amounts as templates for each fish species, and 11 pairs of primers were selected for PCR verification at 30 SNP sites (Table S5). The PCR amplification and 1.5% agarose gel electrophoresis were used to detect the 11 pairs of primers, and a single band with the expected size was produced (Figure 3). The PCR products validated by second-generation sequencing showed that there are 16 sites with differences between BSB and GBSB, which can clearly distinguish different genotypes (Figure 4). There were 5 transversions and 11 transitions, with C/T being the most common (Figure 5). The names of the locus genes, chromosomes, positions, and mutation information are shown in the table. Among the 16 sites, 8 are located in the 3′ UTR, while the remaining 8 loci are located in the coding region of the gene, resulting in 2 synonymous mutations and 6 nonsynonymous mutations. All the non-synonymous mutations were missense mutations (Table 2). For these 16 sites, PCR was performed on 20 individuals from 2 groups, and sequencing was used to verify the genotyping (Figure 6).

### 3.3. Functional Analysis of Core SNP Sites Sequences

**Figure 7:**
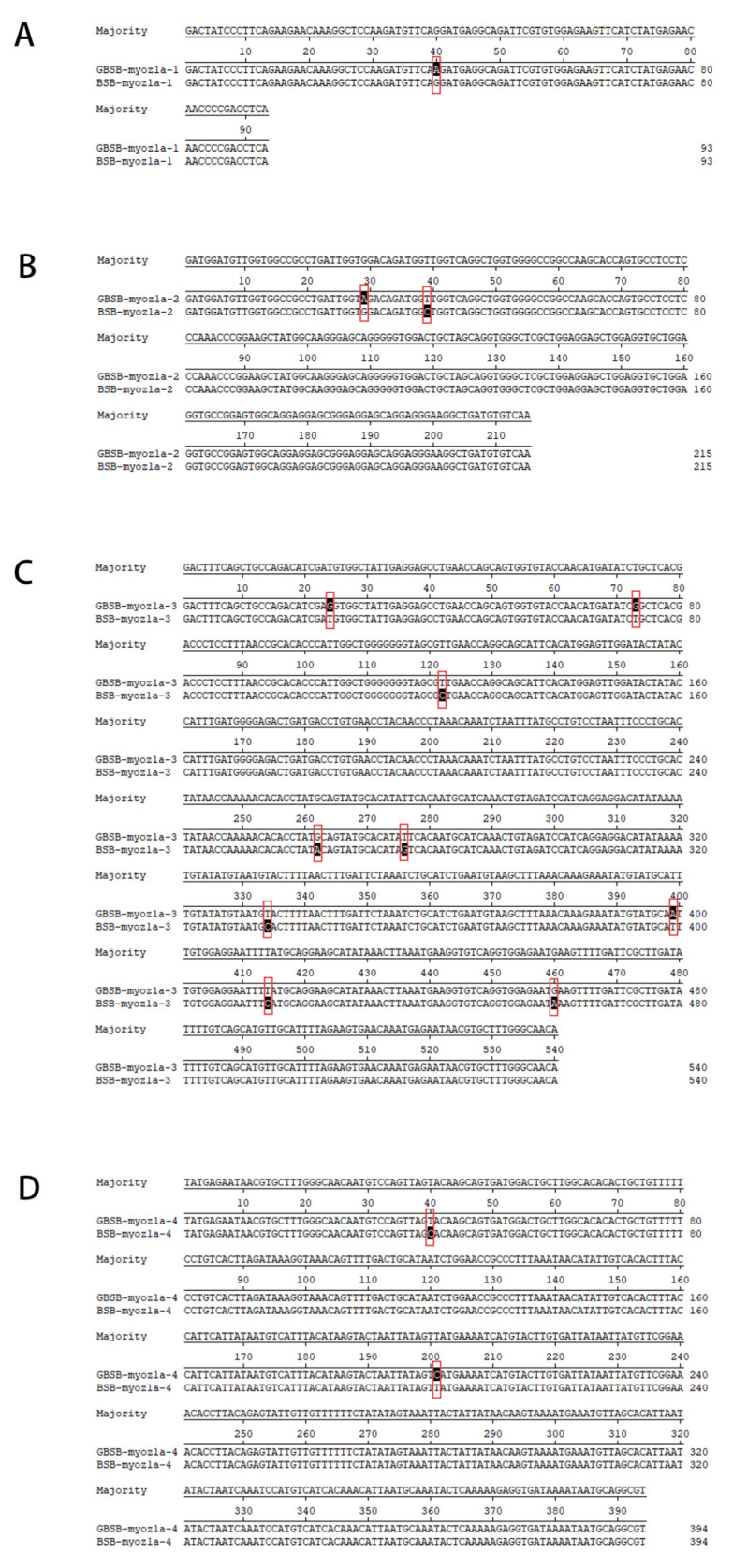
Sequence alignment results of SNP mutation sites corresponding to the GBSB *Myoz1a* gene. (**A**) SNP-10-14178943 (**B**) SNP-10-14179308, SNP-10-14179318, SNP-10-14179486 (**C**) SNP-10-14180581, SNP-10-14180630, SNP-10-14180679, SNP-10-14180819, SNP-10-14180833, SNP-10-14180891, SNP-10-14180956, SNP-10-14180971, SNP-10-14181017 (**D**) SNP-10-14181110, SNP-10-14181271. The differential positions of key SNP loci were marked with red boxes. SNP loci are clustered in the *Myoz1a* gene, which may form a haplotype module. SNP in the 3′ UTR region may regulate gene expression by affecting miRNA binding or mRNA stability.

The SNP sites in the sequencing results were compared by DNAman. Some of the sequence comparison results are shown in Figure 7. Functional annotation of the sequences corresponding to the 16 polymorphic SNP loci successfully screened was performed using NCBI’s BlastP and UniProt, and all 16 SNP loci sequences had clear and valid functional annotations. The open reading frame was inferred using the ORF finder and compared against the NCBI database to ensure the correctness of the ORF selection and determine the position of the SNP sites in the genetic codon. The results showed that the 16 SNPs were distributed in *actn3a* (alpha-actinin skeletal muscle isoform 3a), *myoz1a* (*myozenin 1a*), and *eef2l2* (eukaryotic translation elongation factor 2, like 2), respectively (Table 2).

### 3.4. Analysis of Core SNP Sites Polymorphism

The obtained genotype data were analyzed using POPGEN32 (version 1.32). The results showed that the heterozygosity (H) for 16 SNP loci ranged from 0.1667 to 0.4933. The number of effective alleles (Ne) ranged from 1.2000 to 1.9737. The minimum allele frequency (MAF) ranged from 0.0833 to 0.3333. The polymorphism information content (PIC) analysis of GBSB revealed that the PIC values for the 16 loci ranged from 0 to 0.3689, with 1 locus showing complete monostability (PIC = 0) and the remaining 15 loci showing moderate polymorphism (0.25 < PIC < 0.5) [35]. Through the Hardy–Weinberg equilibrium test, the results showed that only the SNP-18-15442468 site (*p* < 0.05) did not conform to Hardy–Weinberg equilibrium (Table 3). The above analysis proved that most SNP sites obtained could be used for genetic analysis of GBSB.

**Figure 8:**
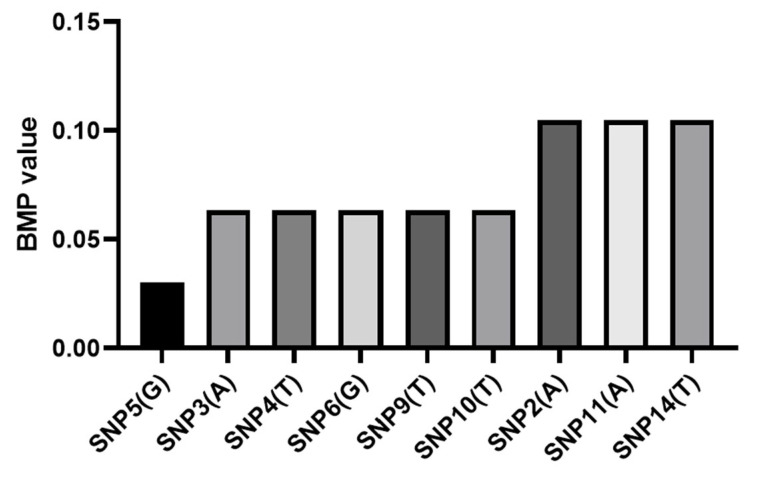
The probability of detecting GBSB-specific genotypes at 9 sites in GBSB. The combined use of multiple markers can significantly reduce the probability of misjudgment and reflect the importance of marker combination.

Among the 15 candidate sites, nine sites, SNP-10-14178943, SNP-10-14179308, SNP-10-14179318, SNP-10-14180581, SNP-10-14180630, SNP-10-14180833, SNP-10-14180891, SNP-10-14180956, and SNP-10-14181110, showed high polymorphism with both homozygotes and heterozygotes in GBSB, while they were conservative in BSB, with only one homozygote. These nine markers can be used for the identification of GBSB and its parent BSB. When the GBSB-specific alleles at these sites are detected, the breeding mating probability (BMP) used for a single marker is shown in Table 4 and Figure 8. The BMP of all 9 markers used in combination ranged from 0.030 to 0.105. The probability of variety coupling can be reduced by using multiple markers together. The markers were sorted according to their ability to distinguish, and the coupling probability of variety identification was calculated when different numbers of markers were combined. With an increasing number of markers, the coupling probability value of the identification of the two species decreases continuously. When the 9 SNPs with the best discrimination ability were used, the MP value was only 3.60765 × 10^−11^, that is, the probability of identification error of the two species was not more than 3.60765 × 10^−11^ (Table 5).

## 4. Discussion

SNP differential analysis, as one of the tools for accurately identifying the relationship between gynogenesis offspring and maternal lineage, has been extensively screened from omics databases. In order to develop GBSB SNP markers, a large number of SNP sites were identified from the transcriptome sequencing data of BSB and GBSB. A total of 30 SNPs were selected that were specific to GBSG and related to genes involved in muscle growth, protein synthesis, and glycolysis. Through multi-sample PCR detection and sequencing analysis, 16 SNPs that showed stable differences in GBSB and BSB were obtained, which were distributed in the genes *actn3a*, *myoz1a*, and *eef2l2*. The polymorphism analysis of 16 SNP sites showed that 9 SNP sites were polymorphic in GBSB, which could be used to identify GBSB and its female parent, BSB.

Heterozygosity reflects the degree of genetic consistency, and polymorphic information content is an indicator to measure polymorphism. The higher the heterozygosity of a population, the lower the degree of genetic consistency of the population, the greater the genetic variation in the population, and the higher the genetic diversity of the population [36,37]. The greater the polymorphic information content, the greater the proportion of heterozygotes at the loci, and the richer the genetic information provided [38]. In the development of molecular markers related to the diet and growth of bigmouth bass, two identified SNP loci exhibited high polymorphism with an average population heterozygosity of 0.4572 [39]. This indicates that the breeding population used by the research team demonstrates significant genetic heterogeneity and rich genetic diversity, suggesting potential for further breeding to enhance population uniformity and improve various production traits [39]. The analysis revealed that the polymorphism information content (PIC) of GBSB SNP loci ranged from 0.2547 to 0.3689 (excluding SNP-18-15442468), indicating moderate polymorphism (0.25 < PIC < 0.5). The heterozygosity (H) distribution ranged from 0.1667 to 0.4933. Notably, when fully homozygous SNP loci were excluded, the PIC values for BSB remained within the same range (0.2547–0.3689), demonstrating comparable genetic diversity levels between GBSB and BSB. Because SNP is marked as a double allele form, each locus carries less polymorphic information, so their theoretical maximum PIC value is lower than that of other molecular markers [40]. The results showed that only the SNP-18-15442468 site (*p* < 0.05) did not conform to the Hardy–Weinberg equilibrium. This deviation may be attributed to selection pressure, population structure, or the gynogenetic breeding history of GBSB. Nevertheless, this deviation does not compromise the SNP marker’s utility for germplasm identification.

Among the 9 SNP sites identified in the final results, the BMP of a single marker was 0.030~0.105. Taking SNP5, which has the smallest coupling probability, as an example, there is only one homozygote “TT” in the BSB population, while GBSB has both homozygote “GG” and heterozygote “TG”, that is, allele “G” is unique to GBSB. If the unknown sample is detected with the “G” allele on the labeled SNP5, it is determined that the sample is GBSB, and the BMP is 0.030. That is, when the SNP5 marker is used alone to identify BSB and GBSB, only 3 individuals are misjudged for every 100 individuals identified. In practical application, using only a single marker is far from meeting the requirements of population number and accuracy in variety identification. In order to achieve a better identification effect, it is usually necessary to increase the number of markers [41,42]. This study identified the unique alleles of GBSB to distinguish it from its parent, BSB. In practical breeding applications, a minimum sample size of 30 individuals per population is recommended to ensure reliable allele frequency estimation. The BMP can be reduced by using multiple markers together. With the increase in the number of markers, the MP value of the identification of two varieties decreases continuously. When four or more SNP markers are used, different BSB and GBSB groups can be identified accurately.

In previous studies, some SNP sites have been reported to be associated with growth. In the largemouth bass, two SNP sites were identified from MSTN, and there was a significant association between the diploids of D2 and D5 and five growth traits [43]. In mandarin fish, four loci (*G1-G3* and *GH-AG*) were identified from the *GH* gene, which showed significant correlation with growth traits [44]. In cattle, four new SNPs and their associated haplotypes were identified from the *FBXO32* gene, two of which were significantly associated with body length [45]. Luo et al. showed that 5 SNPs were identified in *Myoz1* from both Avian and Yellow Bantam chickens, and each had 3 significant effects on some carcass and meat quality traits [46]. The *myoz1* gene encodes a protein that is specifically expressed in muscle tissue and belongs to the actin-binding protein family (calsarcin family) [47]. As a “molecular anchor” of the Z disk, this protein stabilizes the structure of the muscle segment and regulates the signaling pathway of calcineurin, which affects muscle development, contraction function, and metabolic adaptation [48]. However, the molecular markers associated with muscle growth in Myoz1 SNPs have not been reported in fish. In our study, 9 SNP markers identified in GBSB were located on the *Myoz1a* gene. Among the 9 SNP sites, 5 are non-synonymous cSNPs (non-synonymous cSNPs), meaning changes in the base sequence can alter the translated protein sequence, thereby affecting the protein’s function [49]. Additionally, 4 sites are located in the 3′ UTR region of the gene; SNPs in the 3′UTR may influence gene expression regulation, though this requires experimental validation [50,51]. Therefore, the results of this study suggest that the obtained SNPs may modify GBSB growth through muscle growth-related gene *myoz1a*. While these SNPs are located in a muscle development-related gene, their functional impact on growth remains to be validated through expression analysis or gene editing in future studies. It should be noted that this study is based on transcriptome data, which may not capture SNPs in non-coding regions. Additionally, all samples were from a single breeding base, which may limit the applicability of these markers to other geographical populations.

In conclusion, nine SNPs were obtained from GBSB, and four to nine SNPs can be used to identify different groups of BSB and GBSB. This study provides a novel marker set and method for the rapid and accurate identification of GBSB and its parent BSB, which is conducive to improving the efficiency and reliability of breeding work.

## 5. Conclusions

This study successfully developed SNP markers to distinguish gynogenetic blunt snout bream (GBSB) from its maternal parent (BSB), which are morphologically identical. Nine polymorphic SNPs located in the *myoz1a* gene were identified and validated. These markers provide a reliable molecular tool for germplasm identification and support marker-assisted breeding in aquaculture.

## Figures and Tables

**Table 1:** Sample resequencing data quality.

Sample ID	Average Re-Sequencing Depth	Number of Reads	Average Sequencing Bases/%
BSB1	6.54×	47,904,234	95.83
BSB2	6.25×	45,894,576	95.81
BSB3	6.34×	46,494,812	96.58
GBSB1	6.26×	45,818,504	96.5
GBSB2	6.31×	46,168,860	96.79
GBSB3	6.52×	47,828,520	95.99

**Table 2:** Detailed information on the SNP site.

SNP No.	Gene Symbol	Chromosome	Location	Mutation Type
1	*actn3a*	18	15,442,468	T/C
2	*myoz1a*	10	14,178,943	G/A
3	*myoz1a*	10	14,179,308	G/A
4	*myoz1a*	10	14,179,318	C/T
5	*myoz1a*	10	14,180,581	T/G
6	*myoz1a*	10	14,180,630	T/G
7	*myoz1a*	10	14,180,679	C/T
8	*myoz1a*	10	14,180,819	A/G
9	*myoz1a*	10	14,180,833	G/T
10	*myoz1a*	10	14,180,891	C/T
11	*myoz1a*	10	14,180,956	T/A
12	*myoz1a*	10	14,180,971	C/T
13	*myoz1a*	10	14,181,017	A/G
14	*myoz1a*	10	14,181,110	C/T
15	*myoz1a*	10	14,181,271	T/C
16	*eef2l2*	4	21,609,175	G/C

**Table 3:** Gene annotation and amino acid mutations of SNPs.

SNP	Feature ID	Annotation	Length of Contig (bp)	ORF	Length of ORF (aa)	Amino Acid	SNP Site
SNP-18-15442468	XM_048167370.1	*actinin alpha 3a*	3240	204-2894	896	Ser 885 Ser TCT → TCC	2858
SNP-10-14178943	XM_048204714.1	*myozenin 1a*	1913	183-1148	321	Arg 73 Lys AGG → AAG	400
SNP-10-14179308						Gly 120 Arg GGA → AGA	540
SNP-10-14179318						Ala 123 Val GCT → GTT	550
SNP-10-14180581						Asp 267 Glu GAT → GAG	983
SNP-10-14180630						Cys 284 Gly TGC → GGC	1032
SNP-10-14180679						Ala 300 Val GCT → GTT	1081
SNP-10-14180819						3′ UTR	1221
SNP-10-14180833							1235
SNP-10-14180891							1293
SNP-10-14180956							1358
SNP-10-14180971							1373
SNP-10-14181017							1419
SNP-10-14181110							1512
SNP-10-14181271							1673
SNP-4-21609175	XM_048188247.1	Eukaryotic translation elongation factor 2, like 2	3199	153-2738	861	Ala 734 Ala GCC → GCG	2354

**Table 4:** Genetic variability at 16 SNPs in GBSB.

SNP	Allele	Genotype Frequency		PIC	N_e_	H	MAF	Hardy–Weinberg Eguiliberum
		BSB	GBSB	GBSB (BSB)				
SNP-18-15442468	T	0.8165	-	0 (0.2547)	1.9348	0.4832	0.3333	χ^2^ = 8.279 (*p* = 0.004)
	C	0.1835	1					
SNP-10-14178943	G	1	0.5774	0.3689 (0)	1.5000	0.3333	0.1667	χ^2^ = 3.215 (*p* = 0.073)
	A	-	0.4226					
SNP-10-14179308	G	1	0.7071	0.3284 (0)	1.3333	0.2500	0.1250	χ^2^ = 2.059 (*p* = 0.151)
	A	-	0.2929					
SNP-10-14179318	C	1	0.7071	0.3284 (0)	1.3333	0.2500	0.1250	χ^2^ = 2.059 (*p* = 0.151)
	T	-	0.2929					
SNP-10-14180581	T	1	0.8165	0.2547 (0)	1.2000	0.1667	0.0833	χ^2^ = 1.212 (*p* = 0.271)
	G	-	0.1835					
SNP-10-14180630	T	1	0.7071	0.3284 (0)	1.3333	0.2500	0.1250	χ^2^ = 2.059 (*p* = 0.151)
	G	-	0.2929					
SNP-10-14180679	C	0.4082	0.7071	0.3284 (0.3664)	1.9737	0.4933	0.3333	χ^2^ = 1.086 (*p* = 0.297)
	T	0.5918	0.2929					
SNP-10-14180819	A	0.9129	0.8165	0.2547 (0.1464)	1.3055	0.2340	0.1250	χ^2^ = 0.238 (*p* = 0.626)
	G	0.0871	0.1835					
SNP-10-14180833	G	1	0.7071	0.3284 (0)	1.3333	0.2500	0.1250	χ^2^ = 2.059 (*p* = 0.151)
	T	-	0.2929					
SNP-10-14180891	C	1	0.7071	0.3284 (0)	1.3333	0.2500	0.1250	χ^2^ = 2.059 (*p* = 0.151)
	T	-	0.2929					
SNP-10-14180956	T	1	0.5774	0.3689 (0)	1.5000	0.3333	0.1667	χ^2^ = 3.215 (*p* = 0.073)
	A	-	0.4226					
SNP-10-14180971	C	0.8165	0.8165	0.2547 (0.2547)	1.4279	0.2997	0.1667	χ^2^ = 0 (*p* = 1)
	T	0.1835	0.1835					
SNP-10-14181017	A	0.5774	0.7071	0.3284 (0.3689)	1.8503	0.4595	0.2917	χ^2^ = 0.220 (*p* = 0.639)
	G	0.4226	0.2929					
SNP-10-14181110	C	1	0.5774	0.3689 (0)	1.5000	0.3333	0.1667	χ^2^ = 3.215 (*p* = 0.073)
	T	-	0.4226					
SNP-10-14181271	T	0.7071	0.7071	0.3284 (0.3284)	1.7071	0.4142	0.2500	χ^2^ = 0 (*p* = 1)
	C	0.2929	0.2929					
SNP-4-21609175	G	0.5774	0.8165	0.2547 (0.3689)	1.7314	0.4224	0.2500	χ^2^ = 0.812 (*p* = 0.367)
	C	0.4226	0.1835					

PIC: Polymorphism information content; N_e_: Effective number of alleles; H: Heterozygosity; MAF: Minor allele frequency.

**Table 5:** The probability of detecting GBSB-specific genotypes at a single locus in GBSB.

SNP	SNP5	SNP3	SNP4	SNP6	SNP9	SNP10	SNP2	SNP11	SNP14
BMP	0.030	0.063	0.063	0.063	0.063	0.063	0.105	0.105	0.105

## Data Availability

The data are contained within the article. Further inquiries can be directed to the corresponding authors.

## Supplementary Materials

The following supporting information can be downloaded at: https://www.mdpi.com/article/10.3390/biology15020188/s1, Table S1: Distribution of SNP mutation types; Table S2: Distribution of SNP region types; Table S3: Distribution of SNP impact types; Table S4: Details of screening for specific SNPs in GBSB; Table S5: Primer information of the 30 SNP markers.
